# A river runs through it: The causes, consequences, and management of intraspecific diversity in river networks

**DOI:** 10.1111/eva.12941

**Published:** 2020-03-17

**Authors:** Simon Blanchet, Jérôme G. Prunier, Ivan Paz‐Vinas, Keoni Saint‐Pé, Olivier Rey, Allan Raffard, Eglantine Mathieu‐Bégné, Géraldine Loot, Lisa Fourtune, Vincent Dubut

**Affiliations:** ^1^ Centre National pour la Recherche Scientifique Station d'Écologie Théorique et Expérimentale du CNRS à Moulis Université Toulouse III Paul Sabatier UMR‐5321 Moulis France; ^2^ Centre National pour la Recherche Scientifique Laboratoire Evolution & Diversité Biologique Institut de Recherche pour le Développement Université Toulouse III Paul Sabatier UMR‐5174 EDB Toulouse France; ^3^ Laboratoire Ecologie Fonctionnelle et Environnement Université de Toulouse UPS CNRS INP UMR‐5245 ECOLAB Toulouse France; ^4^ IHPE Univ. Montpellier CNRS Ifremer Univ. Perpignan Via Domitia Perpignan France; ^5^ PEIRENE EA 7500 Université de Limoges Limoges France; ^6^ Aix Marseille Université CNRS IRD Avignon Université IMBE Marseille France

**Keywords:** conservation genetics, dendritic networks, eco‐evolutionary dynamics, ecosystem services, intraspecific diversity

## Abstract

Rivers are fascinating ecosystems in which the eco‐evolutionary dynamics of organisms are constrained by particular features, and biologists have developed a wealth of knowledge about freshwater biodiversity patterns. Over the last 10 years, our group used a holistic approach to contribute to this knowledge by focusing on the causes and consequences of intraspecific diversity in rivers. We conducted empirical works on temperate permanent rivers from southern France, and we broadened the scope of our findings using experiments, meta‐analyses, and simulations. We demonstrated that intraspecific (genetic) diversity follows a spatial pattern (downstream increase in diversity) that is repeatable across taxa (from plants to vertebrates) and river systems. This pattern can result from interactive processes that we teased apart using appropriate simulation approaches. We further experimentally showed that intraspecific diversity matters for the functioning of river ecosystems. It indeed affects not only community dynamics, but also key ecosystem functions such as litter degradation. This means that losing intraspecific diversity in rivers can yield major ecological effects. Our work on the impact of multiple human stressors on intraspecific diversity revealed that—in the studied river systems—stocking of domestic (fish) strains strongly and consistently alters natural spatial patterns of diversity. It also highlighted the need for specific analytical tools to tease apart spurious from actual relationships in the wild. Finally, we developed original conservation strategies at the basin scale based on the systematic conservation planning framework that appeared pertinent for preserving intraspecific diversity in rivers. We identified several important research avenues that should further facilitate our understanding of patterns of local adaptation in rivers, the identification of processes sustaining intraspecific biodiversity–ecosystem function relationships, and the setting of reliable conservation plans.

## 1 INTRODUCTION

Rivers are at the heart of humans' life. They have been central to the development of human societies: They aggregate humans and have set the development of most villages and cities around the world. Rivers indeed provide essential resources and services for our well‐being, and have always been used by humans as colonization pathways (Solomon, 2010). Rivers are also attracting as majestic and inspiring landscapes that harbor unique biodiversity, although they paradoxically cover a small area on Earth. For these reasons (and a variety of others), rivers have fascinated many scientists and continue to occupy the mind of most of us.

Rivers are unique ecosystems whose functioning is hardly comparable to any other ecosystem, making them scientifically intriguing. Their spatial arrangement into dendritic arborescence comparable to the hierarchical branching of trees (dendritic ecological networks; Peterson et al., 2013), together with the inherent (sometimes intermittent) downstream‐directed water flow, makes them unique. These two characteristics affect not only the chemical composition and physical architecture of these ecosystems (Benda et al., 2004), but also the ecological and evolutionary dynamics of organisms inhabiting them (Altermatt, 2013; Campbell Grant, Lowe, & Fagan, 2007; Grummer et al., 2019). For instance, dispersal—at least for purely aquatic organisms—is constrained by water corridors and modulated by the water flow and by both natural (e.g., falls) and anthropogenic (e.g., dams) fragmentation, which has consequences for the metapopulation dynamics of organisms and for the maintenance of local (mal‐)adaptation (Fagan, 2002; Fronhofer & Altermatt, 2017; Lytle & Poff, 2004). These features generate unique spatial and temporal patterns of biodiversity (Altermatt, 2013), and a main quest for riverine ecologists is to describe these patterns in natural and altered riverscapes while identifying their underlying processes. Beyond satisfying our scientific curiosity, this quest for key processes is crucial since it represents one of the ways to help preserve rivers and the biodiversity they harbor from the devastating effects of human activities (Tonkin et al., 2019).

Most scientific efforts to describe and understand river biodiversity have been devoted to patterns of species diversity, and the most recent advancements have demonstrated that historical contingencies (e.g., past connectivity among river basins) have shaped large‐scale (regional to continental) patterns of species diversity (Dias et al., 2014; Oberdorff et al., 2019) and that neutral and non‐neutral processes interact to drive the structure of local communities (Altermatt, 2013; Blanchet, Helmus, Brosse, & Grenouillet, 2014; Brown & Swan, 2010; Carrara, Altermatt, Rodriguez‐Iturbe, & Rinaldo, 2012). In particular, the role of dispersal is now acknowledged as central for understanding the distribution of species between and within river basins (reviewed in Tonkin et al., 2018), with strong implications for biodiversity conservation and river management that should be held at the river basin scale rather than according to administrative boundaries.

Patterns of within‐species diversity (intraspecific diversity) have comparatively been much less studied in riverscapes and are rarely the target of global conservation policies (Vernesi et al., 2008), probably because intraspecific diversity is somehow less convenient to describe (but see Beheregaray, Cooke, Chao, & Landguth, 2015; Finn, Bonada, Múrria, & Hughes, 2011; Grummer et al., 2019; Hughes, Schmidt, & Finn, 2009). Nonetheless, this is a key facet of biodiversity, mainly because it is the foundation for species adaptation and diversification and because it is the first component of biodiversity to be altered when the environment changes (Spielman, Brook, & Frankham, 2004). Moreover, intraspecific diversity can represent a significant part of the whole biodiversity in a species assemblage (Siefert et al., 2015), especially in rivers that are naturally species‐poor such as upstream parts of most temperate watersheds (Altermatt, 2013; Blanchet et al., 2014). In these areas, intraspecific diversity *must* be the target of conservation, as it is the main biological source of ecosystem functions and services, and it *must* be preserved in order to maintain ecosystem functionality. It is hence a natural scientific exercise to shift our questionings from inter‐ to intraspecific diversity patterns so as to move toward a unified view of biodiversity structure and dynamics in riverscapes.

**Figure 1:**
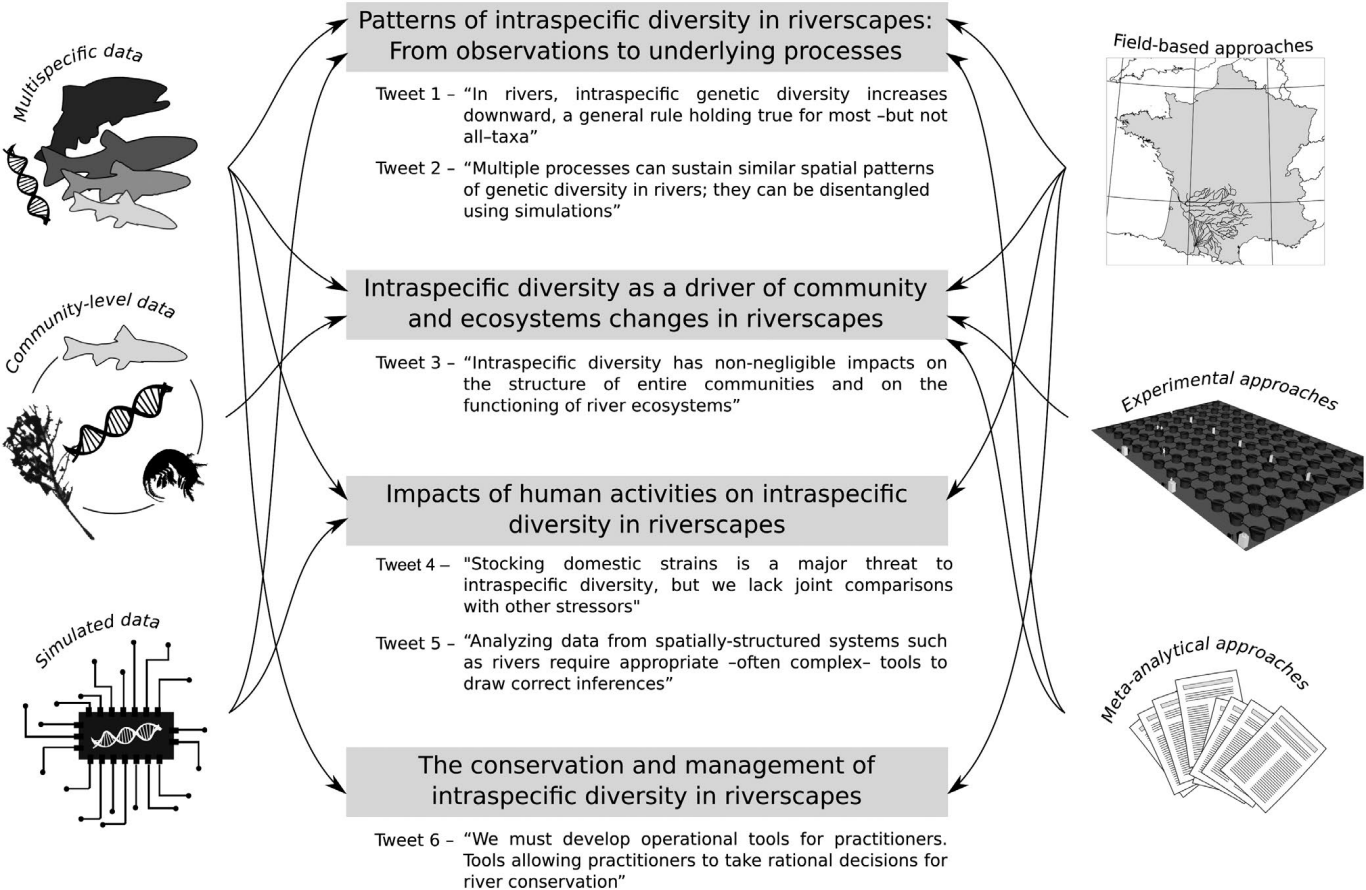
This chart illustrates our main research topics (light gray rectangles) along with our main take‐home messages, in the form of tweets. Side panels indicate the type of data (left) and the type of approaches (right) we considered to investigate each topic

**Figure 2:**
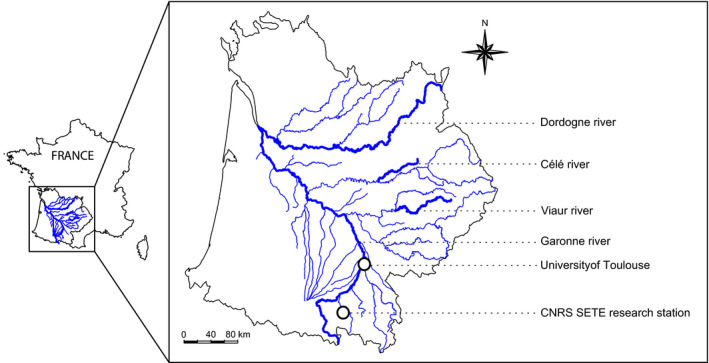
Map of the Garonne–Dordogne River basin in southwestern France, where most of our fieldwork is conducted. The map notably indicates (i) the two main rivers within the basin (the Garonne River and the Dordogne River), (ii) two rivers that we surveyed for up to 15 years (the Viaur River and the Célé River), and (iii) our two host institutions (the University of Toulouse and the SETE laboratory)

We—as a research group—have spent the last 10 years at running through French temperate rivers to catch the intraspecific facet of diversity, to describe the way it is distributed in these particular ecosystems, to understand why it is not uniformly distributed in space, to quantify to which extent human activities alter its distribution, and to propose measures to preserve it. In this retrospective, we reviewed these last 10 years of research. Intraspecific diversity can be measured using various supports, and we have mainly—but not exclusively—focused on genetic diversity, using empirical, experimental, and simulated data (Figure 1). We conducted field‐based approaches, mostly focusing on freshwater fish species within a specific river basin, the Garonne–Dordogne River basin (southwestern France; Figure 2). We focused on this large river basin mainly because its dendritic configuration and the biodiversity it harbors are representative of most permanent temperate watersheds in Europe. Moreover, it is affected by multiple anthropogenic stressors that typically threaten most European rivers [such as fragmentation by old (up to the Middle Age) weirs and recent (>1920) dams, organic and inorganic pollution, non‐native species, or the stocking of hatchery‐born fish], which posit important conservation conundrum for local managers. By working “locally” (i.e., close geographically from our host institutions; Figure 2) we also improve discussions with managers and limit our carbon footprint. We further conducted experimental and meta‐analytical approaches (Figure 1) to broaden the scope of our researches and to generalize our findings beyond the limits of our main field study system. Our first objective was to search for general (i.e., repeatable) patterns of intraspecific genetic diversity. Since such patterns exist across river systems and taxa, we developed original approaches to identify the underlying processes shaping these patterns, as it is mandatory to propose informed and coherent conservation and management actions. Our second objective was to evaluate the ecological importance of such a facet of biodiversity: Can it be a driver of diversity at the species level? Can it—substantially—affect ecosystem functions such as organic matter decomposition or productivity? If yes, this means that intraspecific diversity is more than “just” the fuel for species evolution and adaptation, but that it is also a key driver of the whole ecosystem. As a third objective, we aimed at determining to which extent humans are altering patterns of intraspecific diversity and at identifying which activities are the most impacting for genetic diversity. Finally, we developed—most of the time in close partnership with environmental managers and stakeholders—new ideas and approaches to preserve and manage intraspecific diversity in rivers. For each objective, we will first briefly review what was known at the time we started our research. We will then present what our works brought to the main asked topics, and we will end by presenting what we foresee as key future questions that remain to be addressed.

## 2 PATTERNS OF INTRASPECIFIC DIVERSITY IN RIVERSCAPES: FROM OBSERVATIONS TO UNDERLYING PROCESSES

### 2.1 What did we know?

Biodiversity is heterogeneously distributed in space and time and tends to follow specific (and sometimes repeatable) patterns along geographical or environmental gradients at both the intra‐ and interspecific levels (Lawton, 1996, 1999; Levin, 1992). The latitudinal and altitudinal patterns of interspecific diversity (Gaston, 2000; MacArthur, 1984), or the isolation‐by‐distance pattern of intraspecific diversity (Sexton, Hangartner, & Hoffmann, 2014; Wright, 1943) are among the most recognized spatial patterns of biodiversity. These patterns are determined by the complex interplay between processes shaping biodiversity. For instance, dispersal and environmental filtering affect the taxonomic composition of communities, whereas genetic drift and gene flow drive the genetic diversity of populations. These processes can occur over both ecological and evolutionary timescales, can directly or indirectly be affected by environmental variables, and may shape both *inter‐ and intraspecific* spatial patterns of diversity in comparable ways (Vellend, 2005). From a conservation stance, it is thus necessary to understand how biodiversity is distributed at the landscape scale, what are the relative roles of each process in maintaining patterns of biodiversity, and whether identical or parallel processes affect biodiversity at different organizational levels (e.g., populations, communities).

In riverscapes, spatial biodiversity patterns have long been studied, notably at the metacommunity level (Campbell Grant et al., 2007; Fagan, 2002). Many observational and theoretical studies had, for instance, demonstrated that dendritic connectivity, physical constraints, and landscape features strongly shape spatial patterns of taxonomic diversity in dendritic ecological networks (Altermatt, 2013). At the metacommunity level, for instance, downstream‐biased dispersal due to unidirectional water flow (Muneepeerakul, Bertuzzo, Rinaldo, & Rodriguez‐Iturbe, 2008) and increasing habitat availability/population densities along the upstream–downstream gradient (Muneepeerakul, Weitz, Levin, Rinaldo, & Rodriguez‐Iturbe, 2007) were already known to affect the distribution of taxonomic diversity in rivers, by producing spatial patterns of increasing taxonomic diversity along the upstream–downstream gradient (but see Oberdorff et al. (2019) for a recent counterexample in the Amazon River basin).

At the intraspecific level, empirical studies already suggested 10 years ago that the genetic diversity of freshwater organisms such as fish is frequently structured along the upstream–downstream gradient (Hänfling & Weetman, 2006; Raeymaekers et al., 2008) and most theoretical works explored the effects of asymmetric gene flow due to unidirectional water flow (Fraser, Lippe, & Bernatchez, 2004; Morrissey & de Kerckhove, 2009), dendritic connectivity (Labonne, Ravigne, Parisi, & Gaucherel, 2008), and overland dispersal (Chaput‐Bardy, Fleurant, Lemaire, & Secondi, 2009) on the spatial distribution of genetic diversity in rivers. However, neither empirical nor theoretical studies attempted to generalize findings across spatial and taxonomic scales, which impeded the identification of general patterns of genetic diversity (similar to those observed at the taxonomic level) in dendritic ecological networks. This was surprising given that repeated empirical observations suggested the existence of a general spatial pattern whereby genetic diversity increases along the upstream–downstream gradient (hereafter *downstream increase in genetic diversity*; *DIGD*, Paz‐Vinas, Loot, Stevens, & Blanchet, 2015). This hypothesis, first formulated in 1989 (Ritland, 1989), was indeed verified for various freshwater taxa (Hänfling & Weetman, 2006; Kikuchi, Suzuki, & Sashimura, 2009; Pollux, Luteijn, Van Groenendael, & Ouborg, 2009), but its generalization long remained an open question. It is worth remembering that a decade ago (before the "genomic revolution"), riverscape population and conservation genetics studies mainly focused on one or a few number of species, sampled in one or few rivers (Pauls et al., 2014), hence limiting our capacity to draw general rules concerning spatial patterns of genetic diversity in dendritic ecological networks. Further, the confounding effects of different processes such as downstream‐biased gene flow, historical colonization, and/or anthropogenic fragmentation were thought to be difficult to disentangle using merely descriptive tools (Blanchet, Rey, Etienne, Lek, & Loot, 2010; Raeymaekers et al., 2008)*.*

Advances in molecular techniques (Beheregaray et al., 2015; Pauls et al., 2014), the increasing interest of molecular ecologists for meta‐analytical approaches (Gurevitch, Koricheva, Nakagawa, & Stewart, 2018), the development of simulation tools allowing to generate genetic data under complex eco‐evolutionary scenarios (Hoban, Bertorelle, & Gaggiotti, 2012), and the development of powerful model‐based inference methods such as approximate Bayesian computations (ABC; Beaumont, Zhang, & Balding, 2002) allowed our group (and others; see Beheregaray et al. (2015) for a review in Amazonian fishes) to (a) shift from mono‐ to multispecific population genetics studies, (b) identify general spatial patterns of genetic diversity in riverscapes, (c) disentangle the relative contribution of competing processes shaping spatial patterns of genetic variability, and (d) study temporal genetic variability patterns by conducting continuous‐in‐time genetic monitoring of wild freshwater metapopulations. We present below these lines of research.

### 2.2 What did we learn, and how?

#### 2.2.1 From mono‐ to multispecific genetic diversity assessment

Multispecific genetic diversity assessment constitutes one of the pillars on which our group has built its research over the last decade (see also Beheregaray et al., 2015). We specifically forged notable knowledge by generating a multispecific genetic dataset consisting on the microsatellite genotyping of four sympatric Cyprinid fish species (i.e., *Squalius cephalus*, *Gobio occitaniae*, *Leuciscus burdigalensis*, and *Phoxinus phoxinus*) sampled in two rivers from the Garonne–Dordogne River basin mainly differing by their levels of anthropogenic fragmentation (i.e., the Viaur River, highly fragmented, and the Célé River, less fragmented; see Figure 2; Blanchet et al., 2010). Besides key findings concerning the species‐specificity of genetic responses to habitat fragmentation (see section The impacts of human activities on intraspecific diversity in riverscapes), the analysis of this multispecific genetic dataset allowed us to highlight positive correlations between genetic diversity and distance of sampling sites to the river source akin to those predicted by Ritland in 1989 (i.e., *DIGDs*). We imputed such spatial patterns to downstream‐biased asymmetric gene flow due to unidirectional water flow (Crispo, Bentzen, Reznick, Kinnison, & Hendry, 2005; Fraser et al., 2004), a process that was likely exacerbated in the fragmented river due to upstream‐directed movement impediment by dams and weirs (Hänfling & Weetman, 2006; Raeymaekers et al., 2008; see section The impacts of human activities on intraspecific diversity in riverscapes). Through the joint analysis of this multispecific empirical dataset and of genetic data simulated under linear stepping‐stone models undergoing different degrees of asymmetric gene flow, we further showed that asymmetric gene flow may generate spurious signals of demographic expansion in freshwater species (Paz‐Vinas, Quéméré, Chikhi, Loot, & Blanchet, 2013). These spurious changes were stronger in downstream populations due to the accumulation of genetic diversity resulting from downstream‐biased asymmetric gene flow (*DIGDs*), hence highlighting the potential analytical consequences of river‐specific genetic diversity patterns and underlying processes in the context of demographic change inferences.

Although very useful, the aforementioned multispecific genetic dataset only concerned two temperate rivers, hence limiting our capacity to draw general rules about patterns of genetic diversity and underlying processes at the river basin scale. We tackled this issue by building a broader database focusing on six parapatric freshwater fish species (i.e., *S. cephalus*, *G. occitaniae*, *L. burdigalensis*, *P. phoxinus, Barbatula barbatula,* and *Parachondrostoma toxostoma*) sampled at 92 sites spread over the entire Garonne–Dordogne River basin (Figure 2; Paz‐Vinas et al., 2018). We revealed significant patterns of *DIGD* occurring at the whole river network scale for three out of six species (and a tendency toward this pattern for two other species) when looking at linear relationships between allelic richness and riverine distance from the outlet. Despite this apparent congruence in patterns of intraspecific genetic diversity, we demonstrated that the distribution of genetic diversity in these species was actually idiosyncratic when described using finer spatial models taking into account the topology of the river network (Ver Hoef, Peterson, Clifford, & Shah, 2014). We concluded that observed patterns were likely the product of complex interactions between processes related to the river network structure, to species demographic histories, and to their life‐history traits (Paz‐Vinas et al., 2018).

#### 2.2.2 Toward general spatial patterns of intraspecific genetic diversity

Isolated empirical surveys are informative of particular contexts, but they constitute an invaluable source of information to identify general patterns and processes when they are combined and analyzed as a whole. We took advantage of quantitative reviews, that is, meta‐analyses (Gurevitch et al., 2018), and of the accumulation of published (and available) genetic datasets to *go bigger* (Blanchet, Prunier, & De Kort, 2017) by conducting a large literature survey and meta‐analysis based on 79 metapopulations of different aquatic taxa (plants, fish, arthropods, mollusks, agnates, and amphibians; Paz‐Vinas et al., 2015) sampled in riverscapes from most continents (see table S1 in Paz‐Vinas et al., 2015). Through this exhaustive meta‐analysis, we finally confirmed the existence of a general pattern of *DIGD* repeatable across taxa at the scale of entire dendritic river networks and in rivers strongly varying in their contemporary and historical contexts. In other words, there is a general tendency toward higher genetic diversity in downstream parts of rivers compared with upstream parts, as it is observed for interspecific diversity across taxa. This is—up to our knowledge—one of the first time that published data were synthesized across different taxonomic groups and riverscapes to highlight a repeatable pattern of genetic diversity within a particular ecosystem. Nonetheless (“and because the truth is rarely pure and never simple”; Wilde, 1908), we still found exceptions to this rule: Taxonomic groups whose dispersal is not exclusively dependent on water (i.e., riparian plants that can disperse via nonaquatic propagules and some invertebrates capable of aerial dispersal) did not display significant patterns of *DIGD* (see also Honnay, Jacquemyn, Nackaerts, Breyne, & Van Looy, 2010), highlighting the strong influence of airborne and/or overland dispersal on the distribution of genetic diversity in river systems for these organisms (Campbell Grant et al., 2007; Chaput‐Bardy, Lemaire, Picard, & Secondi, 2008). Surprisingly, we also identified “bell‐shaped” patterns of intraspecific genetic diversity (distributions whereby allelic richness was lower in downstream and upstream parts of river networks compared with intermediate sections) for 10% of the surveyed metapopulations, a pattern that was yet rarely uncovered (but see Alp, Keller, Westram, & Robinson, 2012; Watanabe, Monaghan, & Omura, 2008, for two early empirical observations of bell‐shaped patterns of intraspecific genetic diversity).

#### 2.2.3 Processes driving spatial patterns of intraspecific genetic variability

We fed on ecologists' long‐lasting tradition of coupling empirical observations to theoretical models (Chave, 2013; Gotelli et al., 2009) to disentangle the processes shaping both the *DIGD* and the “bell‐shaped” patterns of intraspecific genetic diversity. We specifically conducted pattern‐oriented genetic data simulations generated under theoretical riverscape models to generate patterns akin to those observed in the aforementioned meta‐analysis. In a first study, we theoretically showed that—everything else being equal—dendritic connectivity per se can generate bell‐shaped patterns of genetic diversity (Paz‐Vinas & Blanchet, 2015). Specifically, highly connected demes (with high *centrality*) situated in intermediate sections of the dendritic network (e.g., confluences) displayed higher allelic richness than low‐connected demes situated at network extremities (i.e., upstream and downstream demes). Inversely, we demonstrated that mean Fst values were higher in low‐connected demes compared with highly connected demes. We imputed these patterns to the proneness of highly connected demes to receive genetically distinct alleles originating from upstream/downstream isolated demes. These results mirrored those from Carrara et al. (2012) who previously highlighted that dispersal along dendritic corridors similarly shapes spatial patterns of taxonomic diversity at the metacommunity level, hence suggesting a theoretical congruency between neutral genetic and species diversity patterns in dendritic riverscapes (see section Intraspecific diversity as a driver of community and ecosystem changes in riverscapes).

The theoretical dendritic models we considered in Paz‐Vinas and Blanchet (2015) were ruled by equal effective deme sizes, symmetric among‐demes migration rates, and no demographic changes over time, being only suitable to highlight the effects of dendricity on spatial patterns of genetic diversity. In a subsequent study (Paz‐Vinas et al., 2015), we extended our simulations to consider downstream‐biased gene flow (Fraser et al., 2004; Morrissey & de Kerckhove, 2009; Paz‐Vinas, et al., 2013), upstream‐directed colonization processes (e.g., after a glacial event; Cyr & Angers, 2012), and/or increasing effective population sizes along the upstream–downstream gradient (due to a downstream increase in habitat availability; Prunier, Dubut, Chikhi, & Blanchet, 2017). We confirmed that these three processes were—independently or interactively—capable of “breaking” the bell‐shaped pattern of genetic diversity arising from dendritic connectivity per se by generating significant patterns of *DIGD* (Paz‐Vinas et al., 2015). In other words, these processes (and their interactions) generated similar genetic footprints when measured as a correlation between distance from the putative river outlet of demes and their allelic richness, hence following a principle of equifinality (Fischer, Maréchaux, & Chave, 2019; Luo et al., 2009), a principle stating that in open systems (here, dendritic metapopulations), a given end state (e.g., the *DIGD* pattern) can be reached by many potential means (e.g., the different processes and interactions we tested). Using machine learning algorithms (i.e., random forests; Breiman, 2001) and ABC model‐choice approaches (Csilléry, François, & Blum, 2012), we nonetheless demonstrated that it is possible to effectively distinguish the processes responsible for an observed *DIGD* in a dendritic river network by considering a set of discriminant summary statistics that differentially react to the considered process(es), hence limiting the uncertainty due to the equifinality problem. For instance, using a subset of studies from the meta‐analysis described above, we demonstrated that the past colonization history of populations was the most likely process to explain most observed patterns of *DIGD* (Paz‐Vinas et al., 2015).

#### 2.2.4 From spatial to temporal patterns of genetic diversity

Spatial patterns of diversity are widely studied, often more than temporal patterns and dynamics. However, an intriguing issue that emerged over the last decades is related to the temporal dynamics of genetic changes, notably for demographically declining populations (Osborne, Carson, & Turner, 2012; Spielman et al., 2004). Most studies assessed genetic changes associated with demographic declines using snapshot approaches, but several authors started to claim that combining continuous‐in‐time genetic monitoring and demographic surveys would be the ideal design to assess the sustainability of wild (and endangered) populations (Habel, Husemann, Finger, Danley, & Zachos, 2014; Schwartz, Luikart, & Waples, 2007; but see Osborne et al., 2012). Demographic and genetic changes could either be linear over time or follow tipping point dynamics, which has very different consequences for the long‐term viability of populations (Hoban et al., 2014). The specific spatial structure of river networks adds a level of complexity since some demes can act as sinks or as sources of genetic diversity, hence influencing the overall metapopulation sustainability over time (Potvin et al., 2017). We tackled this issue by analyzing a 10‐year‐long continuous‐in‐time genetic monitoring survey that we started in 2004 and that we coupled with a demographic survey conducted at the metapopulation scale. This survey was part of a wider research program on a host–parasite interaction and focused on the host, a *L. burdigalensis* metapopulation located in the Viaur River (Figure 2) and that experienced a drastic demographic decline during the survey (Mathieu‐Bégné, Loot, Chevalier, Paz‐Vinas, & Blanchet, 2019). We highlighted a sudden and rapid demographic loss of individuals of about 80% by 2007–2008. Interestingly, even over a period as short as 10 years (which is about five *L. burdigalensis* generations), we were able to detect a loss of genetic diversity associated with this demographic decline. The overall *L. burdigalensis* metapopulation was losing rare alleles and was becoming more inbred, and a genetic bottleneck was emerging over time. In agreement with previous simulation studies, we also found that all indices of genetic diversity were not equally sensitive to demographic changes over time (no change in heterozygosity nor allelic richness were detected; Hoban et al., 2014). Finally, we showed how the spatial arrangement of a metapopulation may impact both demographic and genetic changes over time. In particular, the most downstream deme of the Viaur River metapopulation maintained stable demographic and genetic parameters over time (contrary to all other demes), suggesting that it could act as a source and hence rescue the whole metapopulation (or at least sustain a certain amount of adaptive potential), provided that habitat fragmentation does not impede dispersal between this particular deme and all others. This case study illustrates how thorough knowledge may be gathered over both temporal and spatial scales to propose efficient management plans and actions.

### 2.3 Where should we go?

#### 2.3.1 Exploring specific processes and unexpected patterns of intraspecific genetic diversity

To date, our group has explored through simulations the effects of general processes on spatial patterns of genetic diversity. Although such studies are needed to set clear hypotheses related to patterns of genetic diversity in rivers, they did not consider other specific processes that may occur in some dendritic ecological networks that deviate from those we simulated. This is the case, for instance, for riverscapes experiencing significant levels of water‐flow intermittence, which is typical from many rivers in Mediterranean climates and which might become a rule in many other biogeographical regions due to climate change (Datry, Fritz, & Leigh, 2016). It may also be the case for riverscapes experiencing marked environmental gradients such as Australian riverscapes, where the combined effect of dendritic network structure and hydroclimatic variations has recently been shown to affect patterns of intraspecific genomic diversity in the Murray River rainbowfish (Brauer, Unmack, Smith, Bernatchez, & Beheregaray, 2018). Furthermore, some unexpected patterns such as downstream *decrease* in genetic diversity (Paz‐Vinas et al., 2015) remain poorly understood and deserve further investigation, given that they have also recently been observed at the taxonomic level in fish communities from the Amazon basin (Oberdorff et al., 2019), probably due to the specific biogeographical and geomorphological contexts of the Amazon drainage network, which strikingly differs from that of temperate riverscapes. Further in‐depth empirical and theoretical studies are now required to understand the relative effects of generic versus more specific/regional processes on spatial patterns of intraspecific genetic diversity.

#### 2.3.2 Toward other components of intraspecific diversity

Our group has so far mainly focused on characterizing spatial patterns of neutral genetic diversity and identifying underlying processes such as past demographic events, genetic drift, or gene flow. We are however convinced that the spatial distribution of other facets of intraspecific diversity (e.g., phenotypic, functional, and/or epigenetic variation) must also be assessed to reveal the effects of evolutionary processes acting at different spatial or temporal scales or that cannot be revealed with neutral markers only (e.g., local adaptation; Brauer et al., 2018; Grummer et al., 2019; Putman & Carbone, 2014). We started addressing this issue, for instance, by exploring the role of neutral and adaptive processes in driving phenotypic diversity in freshwater fish (Fourtune, Prunier, Mathieu‐Bégné, et al., 2018; Fourtune, Prunier, Paz‐Vinas, et al., 2018; Raffard, Cucherousset, et al., 2019; see section Intraspecific diversity as a driver of community and ecosystem changes in riverscapes) and by showcasing the importance of epigenetic variation in a biological conservation perspective (Rey et al., 2020; see section The conservation and management of intraspecific diversity in riverscapes). We encourage researchers to join these exciting lines of research by widely exploring spatial patterns of intraspecific diversity sensu lato and their underlying processes at various taxonomic, temporal, and spatial scales.

#### 2.3.3 Toward a community‐wide assessment of intraspecific diversity

We should aim to *go bigger* and characterize spatial patterns of intraspecific diversity at the whole riverscape metacommunity level. Recent advances in high‐throughput sequencing and bioinformatics allow conducting fast biodiversity assessments by massively sequencing molecular markers from multiple species at affordable costs (Delord et al., 2018; Lepais et al., 2019). It is thus becoming possible to rapidly identify species composing riverine communities while estimating the intraspecific (genetic) diversity of all the species forming these communities (e.g., using DNA metabarcoding techniques; Elbrecht, Vamos, Steinke, & Leese, 2018). Although such bioassessment techniques are still under development, their constant improvement and potential future application to riverscapes should greatly increase our knowledge of how biodiversity is distributed in such ecosystems at different hierarchical levels, while improving our capacity to design sound measures for biodiversity conservation at both the intra‐ and interspecific levels.

## 3 INTRASPECIFIC DIVERSITY AS A DRIVER OF COMMUNITY AND ECOSYSTEM CHANGES IN RIVERSCAPES

### 3.1 What did we know?

The influence of interspecific diversity on ecosystems has long been studied to predict the consequences of species loss on ecosystem functions and services (Cardinale et al., 2012; Hooper et al., 2005). In the early 2000s, the first evidence that intraspecific diversity also constitutes a significant driver of community structure and ecosystem functioning has emerged (Whitham et al., 2003). Since then, numerous studies, notably in freshwater systems, have sought to understand eco‐evolutionary dynamics, that is, the links (e.g., feedback loops) between the evolutionary processes modulating patterns of intraspecific diversity and the ecological processes occurring at the population, community, and ecosystem levels (Harmon et al., 2009; Matthews, Aebischer, Sullam, Lundsgaard‐Hansen, & Seehausen, 2016).

At the community level, the species–genetic diversity correlation (hereafter *SGDC*) framework has provided a theoretical foundation for explaining relationships that can be observed between intraspecific genetic diversity and species diversity (Vellend, 2003, 2005). Several mechanisms have been advanced to explain positive *SGDCs*. Especially, genetic diversity within one species may positively affect species diversity of the surrounding community, by promoting the whole *community*‐level stability and by reducing its own extinction risk (Frankham, 2015; Vellend & Geber, 2005). Conversely, genetic diversity might be influenced by species diversity if increased species diversity promotes diversifying selection on non‐neutral genetic diversity. Finally, positive *SGDCs* can also result from coresponses of genetic and species diversity to common environmental factors (Vellend & Geber, 2005). Deciphering the relative (or combined) role of each hypothesis from empirical data remains extremely challenging (Vellend et al., 2014). Intraspecific diversity might also affect the functioning of river ecosystems by shaping the structure of communities at different trophic levels through top‐down and bottom‐up processes and by mediating abiotic parameters (e.g., nutrient recycling; Leitch, Leitch, Trimmer, Guignard, & Woodward, 2014). These effects of intraspecific diversity occurring at the ecosystem level have been mostly studied through the lens of functional traits (i.e., traits affecting ecological processes). Functional traits are linked to the morphology and to the energetic or behavioral status of individuals, which may subsequently modify key ecological features of organisms such as foraging behavior or stoichiometry (i.e., the balance in body nutrient contents; Díaz et al., 2013; Violle et al., 2007). Intraspecific diversity in functional traits (e.g., body size or stoichiometry) has been experimentally shown to be important for key ecosystem functions in rivers including primary production or leaf decomposition (e.g., El‐Sabaawi et al., 2015; Lecerf & Chauvet, 2008). For instance, populations of guppies (*Poecilia reticulata*) that evolved in the presence of predators display specific functional traits (e.g., smaller body size) compared with populations that evolved in the absence of predators (Bassar et al., 2010). These functional “adjustments” in turn affect the community structure (e.g., prey availability) and ultimately affect ecosystem functions (Bassar et al., 2010; Matthews et al., 2011). Further investigations are needed to fully understand the complex relationships between (functional) intraspecific diversity and ecosystem functioning.

Hereafter, we illustrate how our research, based on both experiments and field surveys in the Garonne–Dordogne River basin, improved our knowledge about the links between intraspecific diversity and species diversity in heterogeneous riverscapes, the mechanisms sustaining the effects of intraspecific diversity on ecological processes, and the relative importance of intraspecific diversity in driving community and ecosystem changes compared with key environmental determinants.

### 3.2 What did we learn, and how?

#### 3.2.1 From neutral genetic diversity to functionally important traits

The relationship between genetic and phenotypic diversity is of upmost importance to assess the mechanisms underlying the effects of genetic diversity on community structure and ecological functions. By analogy to the *SGDC* framework, we developed a companion modeling framework (that we named the *genotypic–phenotypic intraspecific diversity correlation*—*GPIDC*—framework) dedicated to understanding spatial variations (and possible covariations) in genetic and phenotypic diversity (Fourtune, Prunier, Mathieu‐Bégné, et al., 2018). We specifically used novel common metrics based on multivariate analyses to describe both genetic and phenotypic diversity on the same statistical basis, so as to facilitate comparisons between both facets of intraspecific diversity. Through its application to data collected at the scale of the Garonne–Dordogne basin (Fourtune, Paz‐Vinas, Loot, Prunier, & Blanchet, 2016; Figure 2), we surprisingly found marked disparities in the spatial distribution of neutral genetic and phenotypic (i.e., morphological traits) diversity for the two studied freshwater fish species (*G. occitaniae* and *P. phoxinus*). Genetic diversity and phenotypic diversity were poorly correlated, and the underlying determinants (at least for phenotypic diversity) were not common across the two species. This suggests that neutral genetic and phenotypic diversity should—in our case study—be considered as independent markers of intraspecific diversity. Interestingly, and although the two species are sympatric and display close ecological requirements, we found contrasting species‐specific phenotypic responses to the abiotic environment. In *G. occitaniae*, we evidenced trait–environment relationships suggesting adaptation or adjustment to local conditions. On the contrary, we did not find any trait–environment relationship in *P. phoxinus*, which could suggest a more "opportunistic" bet‐hedging‐like strategy to cope with environmental variation (Fourtune, Prunier, Paz‐Vinas, et al., 2018).

A subset of *P. phoxinus* populations was further used to investigate the possible relationships between genetic diversity and phenotypic traits that likely matter for ecosystem functioning, that is, functional traits. Using a quantitative genetic approach (Pst‐Fst comparison), we evidenced that functional traits such as body mass, risk‐taking behavior, and metabolic and excretion rates varied among *P. phoxinus* populations occupying rivers differing in their environmental characteristics (e.g., predator abundance and temperature; Raffard, Cucherousset, et al., 2019). Specifically, population differences in body mass, and metabolic and excretion rates were higher than differences expected under the sole influence of genetic drift, suggesting that these trait divergences arose from selection and/or developmental plasticity. On the contrary, genetic drift was important for shaping variability in risk‐taking behavior. These results suggest that *both* adaptive and nonadaptive mechanisms can have ecological consequences on communities and ecosystems, since functional traits are involved in multiple ecological interactions (e.g., predation).

#### 3.2.2 From population genetics to community assembly

Genetic diversity within some single species was found to be correlated with species diversity (positive *SGDC*; Vellend & Geber, 2005). Yet, it is still unclear whether this pattern holds for all species within a community or is restricted to some specific species with particular traits and/or ecological functions. Theory predicts that the strength and sign of *SGDCs* depend upon species characteristics, but empirical studies testing this prediction are scarce, notably because they require a multispecific genetic sampling for both generalities and peculiarities to be identified. Building on a large field survey conducted at the Garonne–Dordogne basin (Figure 2) scale (see the *From mono‐ to multispecific genetic diversity assessment* subsection above), we investigated the relationship between genetic diversity estimated within four parapatric fish species and fish species diversity while accounting for local environmental conditions (Fourtune et al., 2016). We took advantage of “causal” modeling (Fourtune, Prunier, Paz‐Vinas, et al., 2018; Grace, 2006) to unravel the direct and indirect links between environmental variables, species diversity, and intraspecific neutral genetic diversity. Overall, we evidenced that similar processes driven by environmental variables shaped both facets of diversity, hence leading to weak but positive *SGDCs* in all investigated species. For instance, sites at higher altitudes displayed lower levels of species and genetic diversity, because they were located far from the outlet and hence experienced lower levels of immigration from potential downstream sources of diversity. Additionally, we found a direct relationship between genetic and community differentiation between sites, which suggests that genetic drift may influence the structure of metacommunities through morphological, physiological, or behavioral divergence among populations. This work illustrates the benefits of considering intraspecific genetic diversity as a target for conservation planning, as our results suggest (as many others, see Vellend et al., 2014 for a synthesis) that this facet of biodiversity can be a good surrogate of the whole biodiversity observed at the local scale.

#### 3.2.3 From intraspecific diversity to ecosystem functioning

Understanding the relative importance of intraspecific diversity and environmental heterogeneity in shaping ecological processes is important to predict how natural and human‐mediated losses in intraspecific diversity affect ecological dynamics at the ecosystem level (Leigh, Hendry, Vázquez‐Domínguez, & Friesen, 2019; Mimura et al., 2017). Using experiments in aquatic mesocosms, we compared the effects of controlled variations in levels of intraspecific diversity with the effect of an increase in water temperature (Raffard, Cucherousset, Santoul, Gesu, & Blanchet, 2018). We showed that intraspecific diversity among six *P. phoxinus* populations affected community and ecosystem functioning as much as increasing the ecosystem temperature by 2°C. Specifically, we showed that variation in individual body mass and behavior (i.e., the activity) strongly affected the size and abundance of preys consumed by *P. phoxinus*. It is noteworthy that intraspecific diversity and warming acted on ecological dynamics through different mechanisms. Indeed, while intraspecific diversity in fish phenotypes mainly mediated trophic interactions, temperature acted on other ecosystem functions such as litter decomposition rate. We finally demonstrated that the ecological consequences of intraspecific diversity were strong enough to alter the fitness of subsequent generations, leading to *indirect trans‐generational effects* of intraspecific diversity. By comparing the eco‐evolutionary consequences of intraspecific diversity to those of an indisputable environmental driver (temperature), we confirmed that intraspecific diversity really influences ecological dynamics beyond the population level.

### 3.3 Where should we go?

#### 3.3.1 Toward a synthesis of biodiversity–ecosystem function relationships

Understanding the effects of intraspecific diversity on ecological processes is an ever‐growing field of research, and our work supports the claim that this facet of biodiversity is critical for community and ecosystem dynamics in river ecosystems (Raffard, Santoul, Cucherousset, & Blanchet, 2019; Whitham et al., 2003, 2006). Actually, we argue that we are at a tipping point where knowledge gathered on *SGDCs* on the one hand, and biodiversity–ecosystem function relationships on the other hand are important enough to reach a general synthesis on the links between biodiversity (at the intra‐ and interspecific level), environmental variation, and ecosystem functioning. We believe that—although much work remains to be done—next generations should bring together major disciplinary fields such as ecosystem ecology, functional ecology, evolutionary ecology, and molecular ecology to generate a holistic framework of the ecological and evolutionary dynamics of river ecosystems. Such a general framework should be accompanied by complementary studies in the wild. We still poorly know whether intraspecific diversity matters in stochastic natural settings in which environmental variations are not controlled. Hence, novel empirical surveys measuring simultaneously intraspecific diversity, environmental variability, and ecosystem functions have to be performed (Hendry, 2019), and robust statistical methods are to be developed for teasing apart the direct and indirect links among biodiversity components and ecosystem functioning in the wild (e.g., Fourtune, Prunier, Paz‐Vinas, et al., 2018).

#### 3.3.2 Toward a better understanding of the influence of anthropogenic activities on ecosystems

Current global changes add up to natural environmental variations to shape biodiversity patterns and ecosystem functions. Several types of anthropogenic pressures such as climate change, introduction of invasive species, habitat loss and fragmentation, and overharvesting affect intraspecific diversity (Darimont et al., 2009). These human‐induced changes at the intraspecific level may in turn strongly affect community structure and ecosystem functioning (Mimura et al., 2017; Raffard, Santoul, et al., 2019), but this remains virtually unexplored (but see Palkovacs, Kinnison, Correa, Dalton & Hendry, 2012). There is a real need for new fundamental and empirical studies to improve our capacity to predict the effects of human‐induced changes in intraspecific diversity on all components of biodiversity and ecosystems so as to better inform conservation actions.

## 4 THE IMPACTS OF HUMAN ACTIVITIES ON INTRASPECIFIC DIVERSITY IN RIVERSCAPES

### 4.1 What did we know?

Rivers have always been at the core of many socioeconomic issues, and their use for human activities has raised a number of anthropogenic stressors such as overexploitation of fish resources, stocking and/or introduction of non‐native species, water pollution, alteration of flow regimes, destruction, and degradation of habitats and fragmentation (Reid et al., 2018). Two of these stressors have focused our attention in recent years: stocking and riverscape fragmentation.

Stocking is a worldwide management practice used to sustain or to enhance natural populations. It is commonly used in fish and particularly in salmonids to improve recreational angling (Hansen, Fraser, Meier, & Mensberg, 2009). Captive‐bred individuals generally exhibit different characteristics than wild ones, including phenotypic but also genetic features (Christie, Marine, Fox, French, & Blouin, 2016). Releasing them into natural populations is thus a highly concerning issue (Araki & Schmid, 2010; Randi, 2008) because admixture between captive‐bred and wild individuals may influence individual fitness (Geiser & Ferguson, 2001). However, knowledge about the actual impacts of stocking on the spatial distribution of genetic diversity within river networks or on the underlying eco‐evolutionary processes is still required.

The construction of artificial structures in rivers, such as weirs, dams, pipes, and culverts, is another global phenomenon aiming at meeting the need for flow regulation and/ or hydropower supply. In Europe, the construction of these artificial structures dates back to the Middle Ages (12‐15th centuries), but their development is accelerating worldwide in response to the growing demand for non‐fossil energy (e.g., Anderson et al., 2018). Weirs and dams are notably responsible for riverscape fragmentation and are now considered as the most widespread and worrying threat to freshwater ecosystems (Couto & Olden, 2018). Riverscape fragmentation causes habitat patches to be reduced in size and to be isolated from one another. It hence decreases gene flow between populations and favors genetic drift and inbreeding (DiBattista, 2008), with strong expected impacts on patterns of genetic diversity and differentiation. In addition to raising fundamental questions such as the species‐specific response of organisms to the presence of artificial structures, this link between patterns of genetic diversity and riverscape fragmentation also constitutes an opportunity to develop robust operational solutions for the restoration of riverscape functional connectivity. Quantifying riverscape fragmentation from genetic data indeed raises a number of technical and analytical challenges, stemming from the handling of pairwise data (Fourtune, Prunier, Paz‐Vinas, et al., 2018; Prunier, Colyn, Legendre, Nimon, & Flamand, 2015), from the temporal inertia in the setting up of genetic differentiation after the creation of a total barrier to gene flow (Landguth et al., 2010), and from the complex interplay between several evolutionary forces (Jaquiéry, Broquet, Hirzel, Yearsley, & Perrin, 2011).

Stocking and fragmentation are just some of the many human‐induced stressors that can affect patterns of intraspecific diversity in rivers. These numerous anthropogenic stressors often interact with each other and with natural environmental gradients to generate complex eco‐evolutionary dynamics, with intricate direct and indirect relationships, and strong collinearity patterns that are to be disentangled for proper conservation planning. Robust analytical procedures are thus needed to decipher the relative contribution of each stressor to the variability in genetic diversity.

### 4.2 What did we learn, and how?

#### 4.2.1 Stocking as a key determinant of intraspecific diversity

Given the intensity of stocking practices in freshwater systems, we routinely investigate the influence of stocking on patterns of intraspecific genetic diversity in our researches. We showed in a study of the genetic structure of two Cyprinid freshwater fish species (*P. phoxinus* and *G. occitaniae*) in two French rivers (Viaur and Célé rivers; Figure 2) that stocking was a strong and consistent driver of genetic variability across these two river systems (Prunier, Dubut, Loot, Tudesque, & Blanchet, 2018). Moderately stocked populations experienced an increase not only in both standard allelic richness and private allelic richness, but also in genetic uniqueness through local introgression of non‐native alleles, notably in *G. occitaniae*. Similarly, we found that stocking significantly increased genetic diversity and differentiation in brown trout *Salmo trutta* populations from a snow/rain‐fed river of the Garonne–Dordogne River basin (i.e., the Neste d'Oueil; Saint‐Pé et al., 2018). Because the distribution of allochthonous genotypes introduced during stocking events differed from the distribution of wild ones in that river, stocking also strongly affected spatial patterns of genetic diversity. We notably found an overall downstream decrease in genetic diversity in the brown trout when levels of admixture were null to moderate, contrary to the general expectation of a *DIGD* (Paz‐Vinas et al., 2015), although this pattern was reversed for high levels of admixture. More importantly, this study showed that stocking affected dispersal behavior of admixed individuals and that admixed individuals tended to disperse with a higher propensity and on longer distances, which may entail negative feedbacks on the spread of allochthonous alleles (Saint‐Pé et al., 2018).

#### 4.2.2 Fragmentation: a critical determinant of intraspecific diversity

Habitat fragmentation is another major management issue in rivers, calling for both a thorough understanding of its impacts on patterns of intraspecific diversity and the development of robust operational tools for riverscape connectivity restoration. By assessing the genetic structure of four freshwater fish species (Blanchet et al., 2010), we showed that overall genetic diversity was lower, and overall genetic differentiation was stronger in a fragmented riverscape (the Viaur River) than in a nonfragmented riverscape (the Célé River; Figure 2) exhibiting similar abiotic conditions, in accordance with theoretical predictions. We also showed that species‐specific features such as dispersal ability, movement behavior, and life‐history strategies are important predictors of species vulnerability to fragmentation. Similarly, Prunier et al. (2018) found that the local influences of habitat degradation and fragmentation on patterns of genetic diversity and differentiation were both species‐ and river‐specific, sometimes even varying along the river channel, thus preventing any generalizations and calling for further researches.

Mitigating the negative aftermaths of fragmentation is of crude importance. It is thus essential for environmental managers to have access to precise estimates of the impact of weirs and dams on riverscape functional connectivity. Although the indirect monitoring of functional connectivity using molecular data constitutes a promising approach, it is still plagued with several constraints. For instance, the temporal inertia in the establishment of genetic differentiation after barrier creation makes it particularly difficult to compare the impact of obstacles differing in age or in the effective size of the populations they separate. We developed a standardized index of genetic connectivity (*C*_INDEX_), allowing an absolute and independent assessment of the individual effects of obstacles on connectivity (Prunier et al., 2019). We demonstrated that the *C*_INDEX_, based on the comparison between observed and expected measures of genetic differentiation, allows quantifying genetic effects of fragmentation a few generations (~10 generations) after barrier creation, while allowing valid comparisons among species and obstacles of different ages. The computation of the *C*_INDEX_ requires a minimum amount of fieldwork and genotypic data, and solves some of the difficulties inherent to the study of artificial fragmentation in river systems. This makes the *C*_INDEX_ a promising tool for riverscape connectivity restoration.

#### 4.2.3 Intraspecific diversity in face of multiple stressors

Organisms are generally facing multiple stressors in the wild, and one of our recent objectives was to test whether multiple stressors (such as fragmentation, pollution, and stocking) can interact to affect patterns of genetic diversity. Nonetheless, robust analytical procedures are needed to handle the complexity of environmental and biological data collected in rivers (e.g., to cope with strong collinearity patterns among environmental variables). Multicollinearity among predictors is indeed likely to hamper the interpretation of multiple regression results, increasing the risk of drawing erroneous conclusions. Several approaches may be considered such as the creation of orthogonal synthetic predictors using principal component analyses or the use of commonality analyses (Prunier et al., 2015; Ray‐Mukherjee et al., 2014), the latter allowing the joint estimate of both the unique and the shared effects of collinear variables. To properly identify the natural and (multiple) anthropogenic drivers of genetic diversity in two freshwater fish species (*P. phoxinus* and *G. occitaniae*) from two distinct rivers, we designed a generalizable analytical framework based on an AIC‐based model selection coupling the creation of meaningful thematic predictors using principal component analyses and the filtering of thematic predictors at three different steps through selection criteria based on commonality analyses (Prunier et al., 2018). We were hence able to quantify the unique contribution of natural features and anthropogenic stressors to the variance in genetic diversity in these two fish species, showing that the contribution of the network structure was 1.8 times higher than the contribution of anthropogenic stressors including pollution, stocking, and fragmentation. Among anthropogenic stressors, we found that spatial patterns of genetic diversity in both *P. phoxinus* and *G. occitaniae* were more impacted by stocking than by human‐induced fragmentation (Prunier et al., 2018).

### 4.3 Where should we go?

#### 4.3.1 Bigger

We believe that future researches on the impacts of human activities on the intraspecific facet of biodiversity should be conducted at a “macrogenetic” scale (Blanchet et al., 2017), by making the best of increasing availability of large‐scale and high‐resolution datasets in various taxonomic groups. Future studies on the effects of stocking should, for instance, aim to better understand the eco‐evolutionary mechanisms of change in spatial patterns of genetic diversity and differentiation, through the study of additional species characterized by different and contrasted life‐history traits, within additional rivers showing different morphologies, topologies, and hydrographic characteristics (e.g., intermittent versus permanent rivers; tropical versus temperate rivers). Similarly, further investigation is required to unravel the species‐specific or the trait‐specific response of organisms to riverscape fragmentation with, for instance, a focus on invertebrates, as they deeply differ from fish species in terms of effective population sizes and dispersal strategies (Alp et al., 2012).

#### 4.3.2 Toward informed management actions

To warrant effective management prioritization and proper evaluation of restoration measures, it is crucial that environmental managers have access to precise and robust estimates of the individual impact of weirs and dams on functional connectivity. By introducing the *C*_INDEX_ (Prunier et al., 2019), we aimed to tackle a number of technical issues stemming from the indirect quantification of barrier effects from genetic data, although we readily acknowledge that further developments are still needed to make it a fully operational tool. We notably plan to take into account the role of asymmetric gene flow (Paz‐Vinas et al., 2015) and to improve both spatial and temporal resolutions of the *C*_INDEX_ by considering the use of genomic and epigenetic markers (Rey et al., 2020). We strongly encourage other researchers to build on this groundwork or to follow their own lines of research in order to move toward fully operational conservation measures based on the analysis of intraspecific diversity.

## 5 THE CONSERVATION AND MANAGEMENT OF INTRASPECIFIC DIVERSITY IN RIVERSCAPES

### 5.1 What did we know?

How to optimally maintain and preserve biodiversity in a world in which human and financial resources dedicated to conservation are limited? Systematic conservation planning procedures based on cost‐effectiveness analyses and complementarity among conservation areas have been developed to address this critical issue (Margules & Pressey, 2000). The main objective of systematic conservation planning is to identify optimal numbers of areas best representing a predefined amount of the biodiversity observed at the scale of a landscape and that must be preserved in priority at a minimum cost (Paz‐Vinas et al., 2018). Systematic conservation planning tools have been traditionally used for species conservation (Hermoso, Linke, Prenda, & Possingham, 2011; Hermoso, Ward, & Kennard, 2013) but more rarely for intraspecific diversity (but see Carvalho, Torres, Tarroso, & Velo‐Antón, 2019; Carvalho et al., 2017; Thomassen et al., 2011). Genetic diversity is nonetheless the fuel for evolution, and its conservation is mandatory to preserve the evolutionary potential of species and to maintain ecosystems stability, services, and resilience to global changes (Caballero & García‐Dorado, 2013; Forsman & Wennersten, 2016; Hughes, Inouye, Johnson, Underwood, & Vellend, 2008; Mimura et al., 2017). Preserving genetic diversity is at the core of conservation genetics, a relatively young, still‐maturing discipline that is currently upscaling to conservation genomics (Hunter, Hoban, Bruford, Segelbacher, & Bernatchez, 2018; Primmer, 2009).

For many years, riverscape conservation geneticists have focused most of their efforts on evaluating the evolutionary potential of threatened populations or species by assessing their levels of genetic diversity, structure, and inbreeding (e.g., Lippé, Dumont, & Bernatchez, 2006), by estimating evolutionary‐sound parameters such as effective population sizes or among‐populations gene flow (e.g., Alò & Turner, 2005), and/or by estimating genetic diversity changes in response to environmental, anthropogenic, and/or demographic factors (e.g., Bessert & Ortí, 2007; Raeymaekers, Raeymaekers, Koizumi, Geldof, & Volckaert, 2009). The genetic‐based information generated through these studies has also been widely used to define relevant management units for conservation, by building on concepts such as evolutionary significant units (ESU; Fraser & Bernatchez, 2001; Ryder, 1986) or the “50/500 rule” (Frankham, Briscoe, & Ballou, 2002).

These studies have been undeniably useful for guiding specific riverscape conservation actions over years. However, many of them have only focused on the genetic facet of biodiversity, and the combination of genetic information with other types of data (e.g., demographic information) for conservation purposes received little attention until the early 2010s. Further, genetic criteria have long been ignored in large‐scale freshwater biodiversity reserve designs (in contrast to terrestrial environments, e.g., Thomassen et al., 2011) and this task has been mainly conducted by using data gathered at higher organizational levels (i.e., community/metacommunity scales), with a strong focus on preserving species diversity (Linke, Hermoso, & Januchowski‐Hartley, 2019). The planning of protected areas indeed requires huge biodiversity datasets to precisely inform how biodiversity is distributed in space and time (Hermoso et al., 2013; Linke et al., 2019). Although largely available at the interspecific level, the relative lack of large datasets at the intraspecific genetic level may partly explain why conservation planning has rarely been applied to intraspecific diversity. Recent advances in molecular biology and bioinformatics have yet drastically increased our capacity to compile genetic datasets at large spatial, temporal, and taxonomic scales (Blanchet et al., 2017).

### 5.2 What did we learn, and how?

#### 5.2.1 The usefulness of combining demographic and genetic approaches for conservation

Over the last 10 years, many authors advocated that combining demographic and genetic approaches is particularly relevant to efficiently define management units and prioritize conservation actions (Landguth et al., 2014; Palkovacs et al., 2014). We accordingly combined genetic and demographic datasets to assess the eco‐evolutionary status of wild populations. For instance, we jointly analyzed data from a three‐decade‐long French National demographic survey (Poulet, Beaulaton, & Dembski, 2011) and from a snapshot assessment of genetic diversity in an endemic freshwater fish species (the threatened *P. toxostoma*) from the Garonne–Dordogne River basin (Figure 2) to identify populations exhibiting high eco‐evolutionary extinction risks and to propose conservation actions targeted toward these at‐risk populations (Paz‐Vinas, Comte, et al., 2013). Through multidisciplinary analyses including genetic structure assessment, genetic‐based demographic change inference, species distribution modeling, and demographic trend analyses, we demonstrated that this species underwent a general decrease in effective population sizes over the last two to eight centuries and a significant decrease in its distribution range (13.1%) over the last three decades. We further evidenced ongoing demographic declines in five of the twelve rivers we analyzed. We accordingly drew a series of recommendations for prioritizing conservation actions toward populations exhibiting both signs of recent and significant decreases in abundance and small effective population sizes (Paz‐Vinas, Comte, et al., 2013). As stated above (see section Patterns of intraspecific diversity in riverscapes: From observations to underlying processes), this type of approach (but combining continuous‐in‐time genetic monitoring and long‐term demographic surveys) was also successfully used in *L. burdigalensis* metapopulations to identify a specific population that could be used as a source to rescue the whole collapsing metapopulation, a critical knowledge for future restoration plans. These few studies illustrate how combining multidisciplinary approaches and conducting integrated demogenetic monitoring programs may provide valuable output for improving conservation practices.

#### 5.2.2 Identifying priority areas for the conservation of multispecies intraspecific diversity using systematic conservation planning tools

Systematic conservation planning for intraspecific genetic diversity is often based on ecological surrogates such as species distribution data or environmental and geographical descriptors (Hanson, Rhodes, Riginos, & Fuller, 2017; Hermoso et al., 2016) or on genetic summary statistics (Carvalho et al., 2017, 2019). Diniz‐Filho et al. (2012) demonstrated that directly considering raw microsatellite genotypic data in systematic conservation planning was also very efficient for the conservation of the genetic diversity of a single species. We conducted a study whose objective was to evaluate the potential of systematic conservation planning tools to identify priority conservation areas accounting for the intraspecific genetic diversity of a whole species assemblage (Paz‐Vinas et al., 2018). We used microsatellite genotypic data from six freshwater fish species sampled at the whole Garonne–Dordogne basin scale (see section Patterns of intraspecific diversity in riverscapes: From observations to underlying processes and Figure 2). Four of these species (*S. cephalus, G. occitaniae*, *P. phoxinus*, and *B. barbatula*) are common in the Garonne–Dordogne River basin, whereas two are rare endemic species of particular conservation interest (*L. burdigalensis* and *P. toxostoma*). We used a systematic conservation planning optimization tool (Ball, Possingham, & Watts, 2009) with allelic occurrence data to (a) test the influence of different conservation targets and analytical strategies on conservation solutions (i.e., identified priority areas for conservation of intraspecific genetic diversity), (b) evaluate the surrogacy in priority areas among species for preserving their genetic diversity, and (c) assess whether classical genetic diversity indices can predict priority areas. We demonstrated that systematic conservation planning tools are efficient for identifying priority areas representing a predefined part of the total genetic diversity of a whole landscape. With the notable exception of private allelic richness, traditional genetic diversity indices such as allelic richness and genetic uniqueness poorly predicted priority conservation areas for genetic diversity. We further identified weak surrogacy among priority areas identified for each species, suggesting that conservation solutions were highly species‐specific. We showed nonetheless that conservation areas identified using intraspecific genetic diversity from multiple species are more effective than areas identified using single‐species data or using traditional taxonomic information. This study generated novel and exciting knowledge on how to define priority areas for the conservation of intraspecific genetic diversity using dedicated systematic conservation planning optimization tools.

### 5.3 Where should we go?

#### 5.3.1 Toward conservation epigenetics

By allowing the delineation of “evolutionary conservation units” accounting for species evolutionary history and adaptive potential, genetic approaches largely contributed to the improvement of conservation practices. Nevertheless, these approaches only loosely integrate the short‐term ecological history of organisms. We recently reviewed how epigenetic data could be used in this context (Rey et al., 2020). Epigenetics can be defined as the study of all reversible chemical changes involved in the regulation of gene expression without modifying DNA sequences. Epigenetic variations such as DNA methylations are common, and can be reversible and transmitted over generations. They are partly genetically determined, but may also be significantly influenced by environmental conditions (Feil & Fraga, 2012). We synthesized knowledge about the importance of epigenetic mechanisms in orchestrating fundamental development alternatives in organisms and enabling individuals to respond in real time to selection pressures. We notably highlighted the relevance of epigenetic variations as biomarkers of past and present environmental stress events and biomarkers of physiological conditions of individuals. We also showed how epigenetic data could help document the eco‐evolutionary structuring of wild populations, improve conservation‐oriented translocations, define significant conservation units (e.g., Adaptive Significant Units; Funk, McKay, Hohenlohe, & Allendorf, 2012), and study landscape functional connectivity (Rey et al., 2020). We believe that future research should consider highly integrative and holistic approaches combining demographic, conservation genetics/genomics, and conservation epigenetic approaches to reveal eco‐evolutionary changes occurring in natural populations in response to changing environments at multiple timescales (from immediate to long‐term timescales). Such integrative studies should provide powerful information to inform future conservation practices.

#### 5.3.2 Upscaling systematic conservation planning for improving biodiversity conservation

We believe that systematic conservation planning procedures should now be upscaled to identify priority conservation areas in riverscapes by comprehensively taking into consideration the complex dendritic structure of riverscapes, the most ecological‐ and demographic‐relevant variables, and multifaceted biodiversity metrics (taxonomic, phenotypic, neutral/ adaptive genetic, and epigenetic metrics). Performing such a task will be challenging and will require (a) defining sound conservation targets for each biodiversity level, (b) developing unifying frameworks accounting for variation across biodiversity metrics and scales (e.g., Gaggiotti et al., 2018), (c) weighting the relative importance of each facet of biodiversity from a conservation standpoint (e.g., do we have to allocate more efforts/resources to conserve a particular component of biodiversity or, for a given component, should we favor a particular species or taxonomic group?), and (d) developing tools to forecast the success of conservation solutions at a particular time horizon (e.g., 50 or 100 years) considering global change scenarios (e.g., Carvalho et al., 2019). It is on these conditions that we will be able to define ambitious and effective conservation plans taking into account the whole biodiversity of river ecosystems.

## 6 CONCLUSIONS

For those who are reading these last lines and who had the courage to read the entire story: congratulations, you have gone through 10 years of research, twenty‐one of our scientific papers, dozens of electric‐fishing days, hundreds of pages of responses to referees, thousands of hours of intense and passionate—sometimes boozy—discussion, millions of torn hair trying to solve unsolvable analytical problems… in a word, our teamwork. For those who are reading these last lines but rather skimmed through the entire story: we cannot blame you, but we can synthetize our work on the causes and consequences of intraspecific diversity in rivers for you—and for all others—with, just this once, a selected series of “*take‐home tweets*” (see also Figure 1):

Tweet 1—“*In rivers, intraspecific genetic diversity increases downward, a general rule holding true for most—but not all—taxa*”

Tweet 2—“*Multiple processes can sustain similar spatial patterns of genetic diversity in rivers; they can be disentangled using simulations*”

Tweet 3—“*Intraspecific diversity has non‐negligible impacts on the structure of entire communities and on the functioning of river ecosystems*”

Tweet 4—“*Stocking domestic strains is a major threat to intraspecific diversity, but we lack joint comparisons with other stressors*”

Tweet 5—“*Analyzing data from spatially‐structured systems such as rivers require appropriate—often complex—tools to draw correct inferences*”

Tweet 6—“*We must develop operational tools for practitioners. Tools allowing practitioners to take rational decisions for river conservation*”

Beyond the trendy yet ephemeral side of tweets, these concluding statements result from a long‐term, holistic, and integrative research philosophy of combining approaches (molecular tools, simulations, biostatistics, experiments, large‐scale spatial surveys, long‐term surveys, meta‐analysis, comparative studies) and concepts from various fields of ecology and evolution (molecular ecology, functional ecology, evolutionary ecology, ecosystem ecology); an approach directly influenced by Louis Bernatchez' works (Box 1). We believe that this is a powerful way to globally grasp the complexity of the links between environment, intraspecific diversity, and river functioning. We also argue that this approach is useful—and hopefully efficient—to convince managers and stakeholders that intraspecific diversity of aquatic organisms is important and that specific conservation plans should be developed to maintain this facet of biodiversity, as it has been done for interspecific diversity. Nevertheless, much work remains to be done and we, as well as other research groups, are still actively filling these scientific gaps while satisfying our curiosity. We would like to particularly emphasize the need for future researches to highlight the peculiarities or on the contrary the generalities that can emerge from empirical studies encompassing large spatial, temporal, and taxonomic scales (Blanchet et al., 2017). A particularly intriguing question to answer concern the generalities (or lack of) that may be drawn from comparative riverscape studies along wide gradients including various historical, geographical, and social contexts, that is, cross‐continental comparative studies. We hope that the next generations of scientists will take over this scientific challenge, which we believe to be the key to generate novel and insightful knowledge for understanding intraspecific biodiversity patterns in riverscapes and, by extent, in all kind of landscapes and environments.

Box 1 **Some personal reflections on my career (Simon Blanchet) and my meeting with Louis**

Like most anglers, I am passionate about freshwaters and fish since I am 6–7 years old. I have spent so many hours in the water that it was natural for me to seek for a job that was about freshwaters and fish. Fortunately, in my entourage, a scientist (Dr. Pierre Joly from Lyon University in France) sent me a letter to inform me very thoroughly about the different academic possibilities to accomplish that dream. At that time I was 13 years old, and it was during the past century, when the web was disconnected and when people communicated by writing letters (with the hands, a pen, and a sheet of paper!). This is by far the most important letter I have ever read in my life. I perfectly remember that he was finishing this letter by mentioning that the PhD thesis was the best route (he used the expression “la Voie Royale” in French) for people that are passionate. From that day, I knew that it was the route to follow, and that I just had to be patient (and lucky). And this is the route I followed, and this is on that route that I met Louis. I met Louis at the most important and dangerous part of that route; the PhD project. And this is where I have been lucky. Actually, at this moment (in 2003) I met Dr. Julian J. Dodson, another scientist that I cannot really dissociate from Louis. They both trusted me sufficiently to make me disperse from France to Québec City. And they offered me the PhD project that I was dreaming of since I am 13 years old, something about competitive interactions between native salmon and introduced trout (I am a salmonid addict since I'm 9 years old). Moreover, they gave me the opportunity to develop a side project merging behavioral ecology and molecular ecology, a discipline that I discovered as a Master student and that was making a lot of scientific sense for me. These two personalities brought me a lot (as a Scientist and as a Human), but today is Louis's day so I'll not speak further about the memorable lab' parties I had with Julian and his team. Louis was inspiring for me for two main reasons. The first one is his incredible enthusiasm for research ideas. I remember leaving his office full of positive energy and more than happy about the ideas we discussed. He always had the good words (“C'est génial mec ! Fonces !”) that make you stronger and that give you the feeling that you are not stupid and that you can go ahead. Louis is what we can call “a big name”, and being so positive and enthusiastic with young researchers is not only rare for someone like that, but also highly fortifying. The second reason is that at a moment of my scientific life (after having successfully reached the end of the PhD thesis route and when you become independent and get your first “official” salary), I had doubts. I had doubts about my role as a scientist in our society. And this is by looking back to my years with Louis that I chased my doubts and that I took clear decisions about what I wanted to be –as a Scientist. While I was finishing my PhD thesis (running the last hundreds of meters at the end of this long route), Louis was developing the field of evolutionary applications by launching the journal of the same name (Bernatchez & Tseng, 2008) and (co‐) organizing the summit entitled “Evolutionary Change in Human‐altered Environments” (Smith & Bernatchez, 2008). I had also heard him speaking to fish farmers about the role of evolution for aquaculture, and how evolutionary theories can be applied to social (and economical) problems. I realized that it was possible to merge excellent fundamental science with human needs, and I realized that I did not want to be either a “fundamental” or an “applied” scientist, but rather a “Louis Bernatchez” scientist; someone being able to use theories to invent new ideas for our society and for biodiversity. Finally, there is perhaps a last reason why Louis is inspiring me. A few years ago Louis spent a few days in France. He came to my lab and discussed with my colleagues and students. I had not seen him for 10 years. And he was the same. He had not changed; two hundred scientific papers and –amongst others– a Molecular Ecology prize later, he was the same guy; positively encouraging the colleagues and students, happy to exchange with one each other, with always the good words for everyone. So Louis, happy birthday, thanks a lot and do not change!

## CONFLICT OF INTEREST

None declared.
